# Multilevel and subnational analysis of the predictors of maternity continuum of care completion in Nigeria: a cross-sectional survey

**DOI:** 10.1038/s41598-023-48240-z

**Published:** 2023-11-27

**Authors:** Oyewole K. Oyedele

**Affiliations:** 1 International Research Centre of Excellence, Institute of Human Virology, Nigeria (IHVN), Abuja (FCT), Nigeria; 2 Department of Epidemiology and Medical Statistics, Faculty of Public Health, College of Medicine, University of Ibadan, Ibadan, Nigeria

**Keywords:** Epidemiology, Epidemiology, Statistics

## Abstract

Understanding population discrepancy in maternity continuum of care (CoC) completion, particularly in sub-Saharan Africa is significant for interventional plan to achieve optimal pregnancy outcome and child survival. This study thus investigated the magnitudes, distribution, and drivers of maternity CoC completion in Nigeria. A secondary analysis of 19,474 reproductive age (15–49 years) women with at least a birth (level 1) in 1400 communities (level 2) across 37 states covered in the 2018 cross-sectional survey. Stepwise regression initially identified important variables at 10% cutoff point. Multilevel analysis was performed to determine the likelihood and significance of individual and community factors. Intra-cluster correlation assessed the degree of clustering and deviance statistics identified the optimal model. Only 6.5% of the women completed the CoC. Completion rate is significantly different between communities “4.3% in urban and 2.2% in rural” (χ^2^ = 392.42, *p* < 0.001) and was higher in southern subnational than the north. Education (AOR = 1.61, 95% CI 1.20–2.16), wealth (AOR = 1.73, 95% CI 1.35–2.46), media exposure (AOR = 1.22, 95% CI 1.06–1.40), women deciding own health (AOR = 1.37, 95% CI 1.13–1.66), taking iron drug (AOR = 1.84, 95% CI 1.43–2.35) and at least 2 dose of tetanus-toxoid vaccine during pregnancy (AOR = 1.35, 95% CI 1.02–1.78) are associated individual factors. Rural residency (AOR = 1.84, 95% CI = 1.43–2.35), region (AOR = 1.84, 95% CI 1.43–2.35) and rural population proportion (AOR = 1.84, 95% CI 1.43–2.35) are community predictors of the CoC completion. About 63.2% of the total variation in CoC completion was explained by the community predictors. Magnitude of maternity CoC completion is generally low and below the recommended level in Nigeria. Completion rate in urban is twice rural and more likely in the southern than northern subnational. Women residence and region are harmful and beneficial community drivers respectively. Strengthening women health autonomy, sensitization, and education programs particularly in the rural north are essential to curtail the community disparity and optimize maternity CoC practice.

## Introduction

Maternal continuum of care (CoC) is an integrated model continuation of care initiated from pre-pregnancy till the period of postpartum with the dimension of time and place, towards strengthening maternal, newborn and child health (MNCH)^1,2^. Maternity CoC thus centered around antenatal care (ANC), intrapartum and postnatal care (PNC) services received by women during pregnancy, delivery of fetal and after childbirth respectively^3^. Completing the three essential maternity care are critical to; reduce pregnancy related morbidities and mortalities^3,4^.

Maternal morbidity and mortality hitherto remained a public health problem, particularly in the lower-middle-income countries (LMIC) with high-risk fertility behavior^5–7^. Approximately, 800 women dies from preventable pregnancy and childbirth related complications daily, whereas most of all maternal deaths occur in developing countries, with sub-Saharan Africa (SSA) accounting for two-third^8,9^. However, these maternal and neonatal morbidities and mortalities (especially in LMIC) could have been prevented if women adhere to the WHO recommendation on 4 optimum ANC contacts and the recently recommended 8 ANC contacts following the review^10–12^.

Worldwide, the top four countries “South-Sudan, Chad, Sierra-Leone and Nigeria” with highest maternal mortality ratio (MMR) “1150, 1140, 1140 and 917 per 100,000 livebirths” in 2019 are respectively in SSA^13,14^. Although, the most recent national survey in Nigeria reported a lower MMR of 512 (95% CI 447–578) per 100,000 livebirths, with neonatal (NMR), infant (IFR) and under-5 mortality rate (U5MR) of 39, 67 and 132 per 1000 livebirths in 2018 respectively, while Pregnancy related mortality ratio (PRMR) is 556 per 100,000 livebirths in the last decade^15,16^.

According to the Nigerian Demographic and Health Survey (NDHS), 67% (6% increase from 2013) of women aged 15–49 years received ANC and about 57% had at least four contacts in 2018, deliveries in a healthcare facility and deliveries assisted by SBA increased from 36 and 39% in 2013 to about 40% and 43% respectively in 2018, while PNC increased by 12% in the last decade (from 30% in 2008 to 42% in 2018) for women and is currently 38% for newborns^15–17^. However, the national aggregate obscures the regional and community level estimates. For instance, 17% of newborns in northwest received PNC compared to 72% in southwest. Also, 61% of women in urban received PNC compared to 30% in the rural.

Despite the recent increase, ANC, SBA and PNC coverage in Nigeria still fell short of the recommended 90% and thus slow progress in achieving reduced maternal and newborn mortality^12,14,18,19^. This is because many women in Nigeria and sub-Saharan African countries at large fail to either utilize any of these services or complete the vital maternity CoC but, rather drop out of the care continuum^20–22^. Meanwhile, maternity CoC completion has been identified as a core principle and framework to underpin strategies to save lives of newborns and mothers^23^. After all, independent increase in ANC, SBA and PNC has not resulted to a steep decrease in maternal and neonatal mortality, which will halt the sustainable development goal (SDG-3) targeted towards reducing MMR to less than 70 per 100,000 and NMR to 12 per 1000 livebirths by 2030^24^. Hence the need for MNCH program that incorporated completion of maternity CoC model as evidently devised in Egypt in the 90’s to achieve reduced MMR and NMR^25,26^.

Studies has reported that education, wealth, place of residence, media access among other factors are associated with ANC utilization and underutilization in Nigeria^27–30^. Findings also emphasized on the fact that optimal ANC contacts predicts SBA use and the combination of both as well as maternal age, child size, level of education, household wealth and employment status are determinant of PNC use and non-use in Nigeria^19,21,31–33^. There is dearth of literatures on factors associated with maternity CoC completion in Nigeria. However, maternal and husband’s educational level, locality, wealth and parity has been identified as determinant^18, 22,34^. Similar factors in addition to; women autonomy and accessible distance increase the chance of completing CoC in Ethiopia^2,35,36^. Whereas, listening to radio, birth order and signs of pregnancy complication are additional factors associated with maternity CoC completion in The Gambia^37^.

Although there is depth of studies on independent utilization of ANC, SBA and PNC in Nigeria^12,14,19,21,27,29,31–33,38^. Nevertheless, very few has combined the key maternity care to measure CoC coverage gaps and its determinants along the pathway of pregnancy to post-delivery period^20,22^. While Akinyemi et al. only assessed drop out, Oyedele et al. investigated CoC completion but without considering the disparities in community clusters^20,22^. Also, the subnational distribution of maternity CoC completion in Nigeria is unknown.

Thus, this study uniquely investigated the community differentials in magnitude of maternity gamut of care completion based on WHO recommendations, applied multilevel analysis to account for clustering effect and evaluate demographics and health-seeking predictors of maternity CoC completion. This study answered the following research questions; Any disparity in community prevalence of maternity CoC in Nigeria? What are the individual and community-level predictors of maternity CoC completion? What is the impact of subnational distribution on maternity CoC completion. The study outcome will provide evidence-based statistics/fact to assist in developing community-specific strategy to improve MNCH policy program.

## Methodology

### Study design, data and area

**Figure 1:**
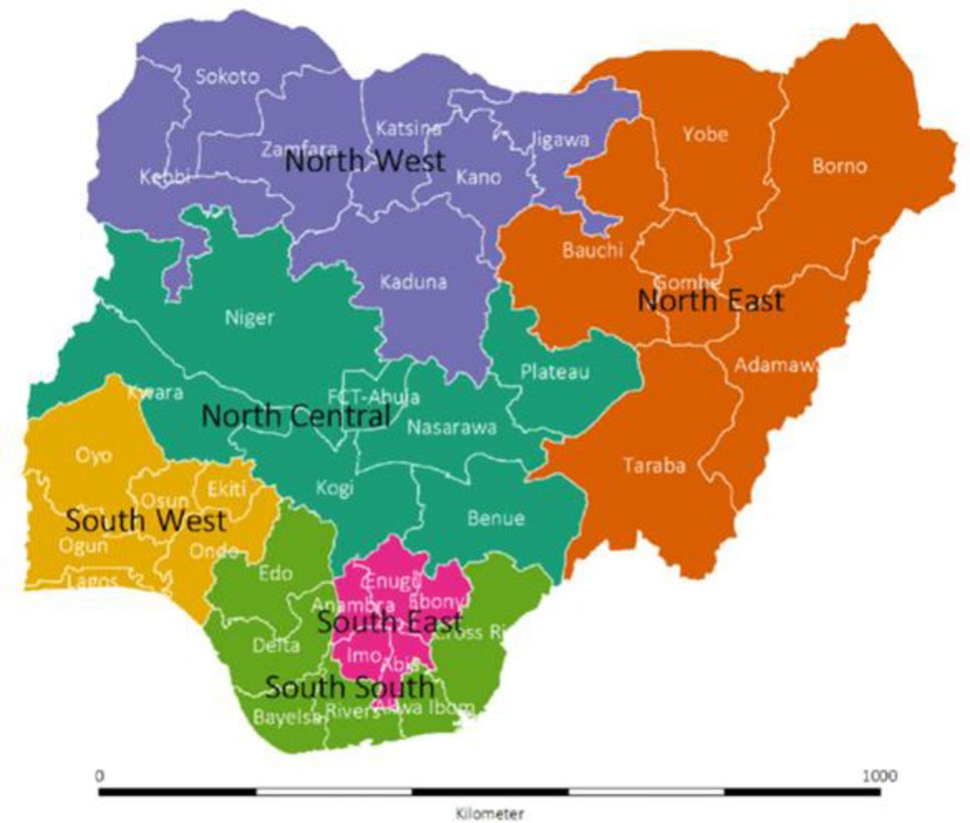
Map of Nigeria showing the 36 states and the federal capital territory (FCT) in the 6 geopolitical zones (Oyedele et al. 2023).

This is a secondary analysis of cross-sectional population-based survey from the nationally representative data of the 2018 Nigerian Demographic and Health Survey (NDHS). The 2018 NDHS is a national representative survey that has been routinely collected in Nigeria since 1990 and often captured respondent across different locations in Nigeria. Administratively, Nigeria is divided into 6 geopolitical zones and each zone or region is subdivided into states and federal capital territory (FCT)^16^. As at 1990, Nigeria comprised of 21 states, 30 states in 1991 and up till now, comprises of 36 states and FCT since the creation of 6 more states in 1996 (Fig. 1)^22^.

### Sampling technique and participants

The 2018 NDHS adopted a two-stage stratified sampling technique using the sampling frame of the Nigeria Population and Housing Census (NPHC) conducted in 2006 by the National Population Commission (NPC). The 36 states and FCT were subdivided into local government Areas (LGA) where urban and rural localities were selected at the first stage sampling (yielding 74 strata). The administrative units were further subdivided into Enumeration Areas (EA) that resulted to the selected 1400 clusters (580 in urban and 820 in rural) called the primary sampling unit (PSU) in the second stage. Thirty households were then selected per cluster (by equal-probability systematic sampling) to make a total of 42,000 households selected and postpartum women of age (15–49 years) were interviewed regarding ANC, SBA and PNC that makes up maternity continuum of care. Information on respondents’ demographic, economic and health indicators were also collected. Comprehensive information on the 2018 NDHS sampling methodology where 41,821 women participants (99% response rate) were recruited has been documented^16^.

### Operational definition

Completion of maternity continuum of care is the outcome variable and, is simply an act of a woman finalizing the main maternal health services provided during pregnancy (Optimal ANC uptake), institutional delivery assisted by skilled births attendants (SBA use) and postnatal care immediately after childbirth till the sixth week of delivery (PNC visit)^22,39^.

### Measure of outcome

CoC is define in this study as a dichotomous response to whether a post-partum woman received ANC in a healthcare facility at least 4 times during pregnancy, childbirths aided by SBA (doctor, nurses or midwifes) and PNC check for the mothers took place after childbirth. Thus, ANC, SBA and PNC are the three key component measures of the outcome variable.

$$CoC\;Completion = \left\{ {\begin{array}{*{20}c} {1,} & {complete \; i.e.\; recieved\; ANC\left( {4 + } \right),\; SBA \;and\; PNC} \\ {0,} & {incomplete \;i.e. \;dropout \;of\; the\; CoC \;at \;least\; once} \\ \end{array} } \right.$$

The outcome was measured according to the WHO recommendations of at least 4 ANC visits and the use of SBA at birth^10,11^. The recently recommended 8 ANC contacts by WHO was not applied to avoid bias from setting stringent rule, since the DHS operationalization was based on birth in the last 5 years using the minimum of 4 ANC contacts, which is yet to even achieve required coverage and also the updated WHO guidelines for 8 ANC contacts were recently implemented after the births of most women respondents in the DHS in Nigeria^16,40^. The measure of PNC for mothers after birth was however due to the fact that most of maternal morbidity (e.g. Postpartum-hemorrhage, Puerperal-sepsis, etc.) and mortality occur in this period and therefore make it a crucial indicator in measuring maternity completion of care and even towards assessing the possible attainment of the SDG-3^8,41^.

### Explanatory variables

Explanatory variables in the study were classified based on individual demographic and autonomy, community, socio-economic, childbirth and mother interaction with healthcare system, similar to classification from previous studies and as a model framework for maternity continuum of care predictors^2,4,35–37,41^. The independent variable categories are also consistent with the variable classification on the DHS and previous literatures on maternity care service^42–46^. Also, the behavioral ecological framework was adopted to classify the independent factor as follows^14,47^.

#### Individual and household-level characteristics

Maternal age (15–24, 25–34, 35–49), level of education (none, primary, secondary, higher), marital status (unmarried, married), partner’s level of education (no education, primary, secondary, higher), religion (catholic/christian, islam, tradintional/other), ethnicity (hausa/fulani, igbo, yoruba, other), getting medical help; permission (not a big problem, big problem), women healthcare decision (partner alone, woman alone, joint decision). Wanted pregnancy (then, later no more), birth order (1, 2, 3, 4 +), provider of ANC (doctor, nurse/midwifery, auxiliary nurse/midwifery, CHEW/CHO, TBA, no one), tetanus toxoid vaccine taken (none, 1, 2+), institutional delivery (no, yes) delivery by CS (no, yes), child sex at birth (male, female), child size at birth (very large, larger than average, average, smaller than average, very small), Wealth status (poor, middle, rich), mass media exposure (no, yes), covered by health insurance (no, yes), medical help-money (not a big problem, big problem).

#### Community-level factors

Place of residence (urban, rural), region (northeast, northwest, northcentral, southeast south-south, southwest), medical help-distance (not a big problem, big problem) Community unemployment (low, average, high), community socio-economic (low, average, high), community rural proportion (low, high).

### Statistical analysis

Basic descriptive analysis was performed and reported in frequency (percentage) for all categorical responses. The frequency and proportion reported was based on women characteristics by the CoC completion status (Yes or No) and thus the supporting bivariate Chi-square statistics was reported. The Pearson Chi-square was reported throughout as none of the expected cell count was < 5. Also, the Phi and Crammer’s V statistics that assessed the relationship between the women CoC completion and the binary/multinomial independent factors were reported respectively.

Further bivariate associations were evaluated using the forward stepwise regression approach such that the empty model (without independent factor) was first fitted, and the individual and community factors are then added in turn. Thus, the factors that significantly increase the − 2LogL at *p* < 0.10 were retained and added to the model and otherwise excluded. This validated the associated factors in the bivariate chi-square analysis as the factors measuring both child-sex and community illiteracy insignificantly add to the − 2logL. Also, identified the variables for inclusion in the multivariable analysis. Hence, the regression coefficients and Wald statistics were reported.

The multilevel (mixed-effect) binary logistic regression assessed the fixed individual-level effect and the random community-level effect. The respective adjusted effect (AOR) was reported for each individual and community factor. Estimate of the intra-class correlation (ICC) was determined from the null model and compared to the fitted full model to obtain the proportion of explained variation in the model which is referred to as the proportional change in variance (PCV). The median odds ratio (MOR) was assessed to evaluate the cluster-level risk of completing the CoC. The likelihood deviance, Akaike and Bayesian statistics compared the null, random, fixed, and full multilevel model fitness. Data management and analysis were conducted using Stata version 17.0 and all statistical tests and analysis were carried out at a 5% level of significance (95% confidence interval). Maps reported in this study were generated from Microsoft 360 (@Microsoft OpenStreetMap). Sampling weights indices included in the NDHS data (Women’s individual weight) were applied and the svyset command was used to adjust for disproportionate sample due to the complexity of the survey design. Multicollinearity was investigated through variance inflation factor (VIF) and controlled by excluding and substituting type of union (VIF > 5.0) with marital status (with weaker correlation coefficients) in the multivariable analysis.

### The multilevel analysis

Multilevel model was fitted at each model stage (model-I, model-II model-III and model-IV) to evaluate enumeration area cluster dependencies in prediction of maternity continuum of care. Model-I is the empty model i.e., intercept only terms, model-II is the individual only term, model-III comprises of the community term while model-IV combined all the terms as a full model^48,49^. The individuals (postpartum women) within households are nested in communities’ enumeration area cluster. Therefore, resulted in a two-level mixed-effect logistic regression with fixed effect at the respondent’s household level and random effect at the community level. The two-level mixed effect model can be expressed as (Level 1, Level 2):

1 $$\ln \left( {\frac{{P_{ij} }}{{1 - P_{ij} }}} \right) = \beta_{0j} + \mathop \sum \limits_{p = 1}^{n} \beta_{\left( p \right)} X_{\left( p \right)ij} + e_{ij}$$

2 $$\beta_{0j} = \alpha_{00} + \tau_{0j}$$

In general, the full multilevel model is produced when Eq. (2) is substituted into (1). Therefore, lead to Eq. (3) below.

3 $$\ln \left( {\frac{{P_{ij} }}{{1 - P_{ij} }}} \right) = \alpha_{00} + \tau_{0j} + \mathop \sum \limits_{p = 1}^{n} \beta_{\left( p \right)ij} X_{ij} + e_{ij}$$

where $\ln \left( {\frac{{P_{ij} }}{{1 - P_{ij} }}} \right)$ is the odds of postpartum women completing the maternity continuum of care, ${\alpha }_{00}$ is the fixed intercept (grand mean), ${\tau }_{0j}$ is the community j random intercept term, ${\beta }_{(p)}$ are the regression coefficients of the independent variables or covariates, ${X}_{ij}$ are the set of individual i and community j predictors, ${e}_{ij}$ is the noise or error term, ${e}_{ij}$ and ${\tau }_{0j}$ are normally, identically and independently distributed with mean 0 and constant variance ${\sigma }^{2}$.

Thus, the ICC measured the degree of clustering based on heterogeneity between clusters i.e., the proportion of total variation in maternity CoC attributable to the community level random-effect component which can be expressed as:

4 $$ICC_{Community} = \frac{{\sigma_{u}^{2} }}{{\sigma_{u}^{2} + \frac{{\pi^{2} }}{3}}} = \frac{{\sigma_{u}^{2} }}{{\sigma_{u}^{2} + \frac{{3.142^{2} }}{3}}}$$

where ${\sigma }_{u}^{2}$ is the between cluster variance (community random-component) and $\left( {\frac{{\pi^{2} }}{3} = 3.291} \right)$ is the within community cluster variance from assumed normally distributed population^49,50^.

The explained variance is computed from the proportional change in variance (PCV) which estimates the difference in variance between the null and the model with at least a factor term (i.e., including covariates) as a ratio of the null model variance.

5 $$PCV = \frac{{\sigma_{0} - \sigma_{i} }}{{\sigma_{0} }}$$

where ${\sigma }_{0}$ is the variance of the null model, ${\sigma }_{i}$ is the variance of observed factor for i is the number of factor or covariates terms in the models i.e., model II, III and IV^21,49,50^.

The median odds ratio (MOR) measures the odds of CoC completion at high-risk cluster relative to the low-risk cluster if two reproductive age women are randomly selected from the two clusters.

6 $$MOR \, = exp^{{\sqrt {2 \times \sigma_{v}^{2} \times \;0.6745 } }} = exp^{{0.95 \sqrt {\sigma_{v}^{2} } }}$$

The fit statistics was assessed based on the likelihood deviance (− 2LL). Akaike (AIC) and Bayes (BIC) Information Criterion were computed from the respective model log likelihood. The model with the least deviance was however adjudged to best predict the CoC completion^49–51^.

### Ethical approval and consent to participate

The study was a secondary analysis and thus relies on the ethical clearance of the DHS which was obtained from the Institutional Review Board (IRB) of Inner City Fund (ICF) International Macro at Fairfax, Virginia, United States, and the country IRB (National Health Research Ethics Committee) in Nigeria. The author was granted access to the DHS data. Written informed consent was obtained from all survey participants prior to data collection. This study adheres to appropriate reporting guidelines and did not involve any conduct of experiment or clinical trial.

## Results

### Women characteristics and association with maternity CoC completion

Table 1 presented the women sample distribution and the bivariate association with maternity CoC completion. Most (48.4%) women are 25–34 years, married (97.1%) and without formal education (46.8%). About 45% (8752) are poor (only 0.8% completed CoC) while 35.1% (with 30.8% incomplete CoC) are rich. Over 51% have had four births (only 2.5% completed CoC) while only 15.5% had one birth. Only 39.5% are exposed to mass media (Table 1). Only 5.1% and 6.1% of women that receive TT (52.5%) and iron drug (69.4%) completed the CoC. About 97% (6% CoC completion rate) had vaginal birth, health insurance only covered 2.3% of the women and only 39.8% had health facility delivery with distance being a problem in 28.3% (5519) (Table 1). About 61.5% reside in rural areas with high rural proportion and poverty rate of 75.3% and 42.9% respectively. All the women factors were associated with CoC completion (ANC4 + SBA + PNC) at *p* < 0.10 except child sex (*p*-value = 0.821) and community illiteracy (*p*-value = 0.931) (Table 1).

**Table 1:** Women distribution and association with maternity continuum of care completion.

Characteristics	Maternity CoC completion (ANC4 + SBA +PNC)			Chi-square	Phi/Crammer’s V	*p*-value
	No n (%)	Yes n (%)	Total n (%)			
Woman’s age				21.44	0.03^ V^	< 0.001
15–24	4429 (22.7)	242 (1.3)	4671 (24.0)			
25–34	8779 (45.1)	643 (3.3)	9422 (48.4)			
35–49	4997 (25.7)	384 (1.9)	5381 (27.6)			
Education				775.22	0.20^ V^	< 0.001
No formal education	8959 (46.0)	155 (0.8)	9114 (46.8)			
Primary	2624 (13.5)	186 (0.9)	2810 (14.4)			
Secondary	5223 (26.8)	639 (3.3)	5862 (30.1)			
Tertiary	1399 (7.2)	289 (1.5)	1688 (8.7)			
Marital status				29.67	0.04^P^	< 0.001
Married	17,700 (90.9)	1205 (6.2)	18,905 (97.1)			
Unmarried	505 (2.6)	64 (0.3)	569 (2.9)			
Partner education				584.74	0.17^ V^	< 0.001
No formal education	7130 (36.6)	87 (0.4)	7217 (37.1)			
Primary	2526 (13.0)	167 (0.9)	2693 (13.8)			
Secondary	5946 (30.5)	638 (3.3)	6584 (33.8)			
Tertiary	2603 (13.4)	377 (1.9)	2980 (15.3)			
Religion				346.95	0.13^ V^	< 0.001
Christianity/catholic	6150 (31.6)	774 (3.9)	6925 (35.6)			
Islam	11,964 (61.4)	491 (2.5)	12,455 (63.9)			
Traditional/other	91 (0.5)	4 (0.1)	94 (0.5)			
Ethnicity				748.84	0.20^ V^	< 0.001
Hausa/fulani	8763 (45.0)	262 (1.3)	9025 (46.3)			
Yoruba	1962 (10.1)	322 (1.7)	2284 (11.7)			
Igbo	1949 (10.0)	359 (1.8)	2308 (11.9)			
Others	5531 (28.4)	326 (1.7)	5857 (30.1)			
Wealth				679.09	0.19^ V^	< 0.001
Poor	8597 (44.1)	155 (0.8)	8752 (44.9)			
Average	3619 (18.6)	273 (1.4)	3892 (20.0)			
Rich	5989 (30.8)	841 (4.3)	6830 (35.1)			
Media exposure				294.64	0.12^P^	< 0.001
No	11,325 (58.2)	457 (2.3)	11,782 (60.5)			
Yes	6880 (35.3)	812 (4.2)	7692 (39.5)			
Wanted pregnancy				40.75	0.05^ V^	< 0.001
Then	16,360 (84.0)	1071 (5.5)	17,431 (89.5)			
Later	1275 (6.6)	145 (0.7)	1420 (7.3)			
No more	570 (2.9)	53 (0.3)	623 (3.2)			
Birth order				52.33	0.05^ V^	< 0.001
1	2760 (14.2)	250 (1.3)	3010 (15.5)			
2	3208 (16.5)	310 (1.6)	3518 (18.1)			
3	2775 (14.2)	211 (1.1)	2986 (15.3)			
4 +	9462 (48.6)	498 (2.5)	9960 (51.1)			
Health insurance				50.23	0.05^P^	< 0.001
No	17,830 (91.6)	1199 (6.1)	19,029 (97.7)			
Yes	375 (1.9)	70 (0.4)	445 (2.3)			
Medical help – permission				25.62	0.04^P^	< 0.001
Not big problem	16,011 (82.2)	1165 (6.0)	17,176 (88.2)			
Big problem	2194 (11.3)	104 (0.5	2298 (11.8)			
Medical help – money				72.01	0.06^P^	< 0.001
Not big problem	9335 (47.9)	814 (4.2)	10,149 (52.1)			
Big problem	8870 (45.6)	455 (2.3)	9325 (47.9)			
ANC provider				697.90	0.19^ V^	< 0.001
Doctor	1037 (5.4)	230 (1.1)	1267 (6.5)			
Nurse/midwifery	10,152 (52.1)	952 (4.9)	11,104 (57.0)			
Auxiliary nurse/midwifery	387 (2,0)	54 (0.3)	441 (2.3)			
CHEW/CHW	1541 (7.9)	20 (0.1)	1561 (8.0)			
TBA/no one	5088 (26.1)	12.3 (0.1)	5101 (26.2)			
Healthcare decider				257.97	0.12^ V^	< 0.001
Partner	11,234 (57.7)	455 (2.3)	11,689 (60.0)			
Respondent	1608 (8.3)	198 (1.0)	1806 (9.3)			
Joint decision	5363 (27.5)	616 (3.2)	5979 (30.7)			
Iron drug taken				326.86	0.13^P^	< 0.001
No	5880 (3.2)	82 (0.4)	5962 (30.6)			
Yes	12,325 (63.3)	1187 (6.1)	13,512 (69.4)			
TT taken				477.25	0.15^ V^	< 0.001
None	5854 (30.1)	65 (0.3)	5919 (30.4)			
1	3128 (16.0)	211 (1.1)	3339 (17.1)			
2 +	9223 (47.4)	993 (5.1)	10,216 (52.5)			
Place of delivery				344.43	0.13^P^	< 0.001
Home	11,250 (57.8)	462 (2.4)	11,712 (60.2)			
Health facility	6955 (35.7)	807 (4.1)	7762 (39.8)			
Mode of delivery				184.22	0.10^P^	< 0.001
Vaginal delivery	17,725 (91.0)	1148 (5.9)	18,873 (96.9)			
Delivery by CS	480 (2.5)	121 (0.6)	601 (3.1)			
Child sex				0.05	0.00^P^	0.821
Male	9325 (47.9)	648 (3.3)	9973 (51.2)			
Female	8880 (45.6)	621 (3.2)	9501 (48.8)			
Child size				9.14	0.02^ V^	0.010
Small	2524 (13.0)	139 (0.7)	2663 (13.7)			
Average	9313 (47.8)	696 (3.6)	10,009 (51.4)			
Large	6368 (32.7)	434 (2.2)	6802 (34.9)			
Place of residence				392.42	0.14^P^	< 0.001
Urban	6650 (31.2)	843 (4.3)	7493 (38.5)			
Rural	11,555 (59.3)	426 (2.2)	11,981 (61.5)			
Geopolitical zone				723.81	0.19^ V^	< 0.001
North central	2571 (13.2)	146 (0.8)	2717 (13.9)			
Northeast	3433 (17.6)	120 (0.6)	3553 (18.2)			
Northwest	7000 (40.0)	202 (1.0)	7202 (37.0)			
Southeast	1511 (7.8)	250 (1.3)	1761 (9.0)			
South-south	1447 (7.4)	158 (0.8)	1605 (8.2)			
Southwest	2242 (11.5)	393 (2.0)	2635 (13.5)			
Distance to medical facility				48.13	0.05^P^	< 0.001
Not a big problem	12,940 (66.5)	1015 (5.2)	13,955 (71.7)			
Big problem	5265 (27.0)	254 (1.3)	5519 (28.3)			
Rural proportion				60.03	0.06^P^	< 0.001
Low	4356 (22.4)	463 (2.4)	4819 (24.7)			
High	13,849 (71.1)	806 (4.1)	14,655 (75.3)			
Community illiteracy				0.1437	0.00^ V^	0.931
Low	2305 (11.8)	146 (0.7)	2451 (12.6)			
Average	7395 (37.9)	535 (2.7)	7930 (40.7)			
High	8505 (43.7)	588 (3.0)	9093 (46.7)			
Community poverty				57.03	0.05^ V^	< 0.001
Low	5283 (27.1)	468 (2.4)	5751 (29.5)			
Average	4976 (25.6)	392 (2.0)	5368 (27.6)			
High	7946 (40.8)	409 (2.1)	8355 (42.9)			
Total	18,205 (93.5)	1269 (6.5)	19,474 (100.0)			

*ANC* Antenatal Care, *CHEW* Community Health Extension Worker, *CHW* Community Health Worker, *TBA* Traditional Birth Attendants, *TT* Tetanus Toxoid, *CS* Caesarian Section, ^P^ Phi Statistic, ^V^ Crammer’s V Statistic.

### Coverage of maternity continuum of care completion

**Figure 2:**
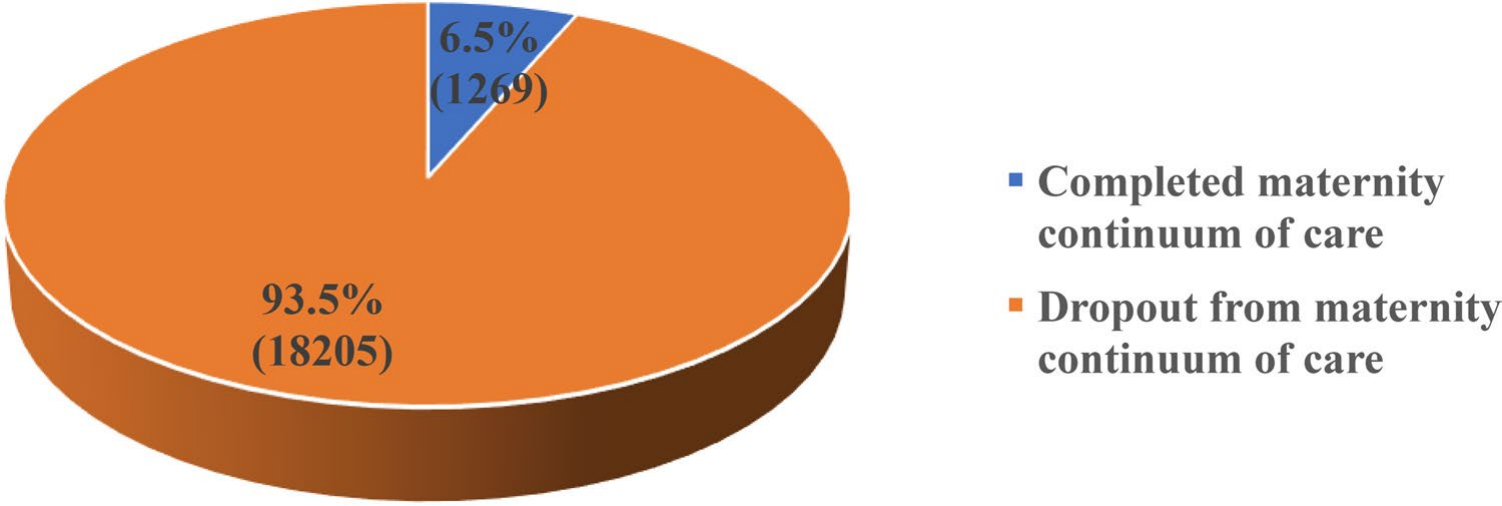
Coverage of Maternity Continuum of Care Completion and Dropout among Women of Reproductive age (15–49 years).

The coverage of maternity CoC completion is shown in Fig. 2. Overall, 6.5% (1269) of the women completed the maternity CoC while as high as 93.5% (59.3% in rural and 34.2% in urban) did not complete the maternity gamut of care (Fig. 2).

### Community prevalence of maternity continuum of care

**Figure 3:**
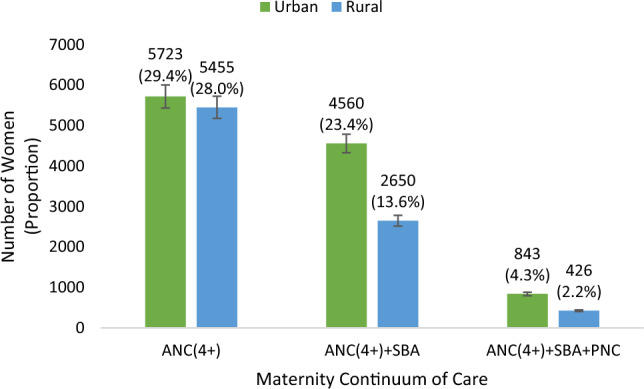
Community Prevalence of Maternity Continuum of Care Completion among Women of Reproductive age (15–49 years).

Figure 3 shows prevalence of maternity CoC by urban and rural communities. Prevalence of optimal ANC is different by 1.4% between urban and rural (Fig. 3). Less than half (13.6%) of the women who had optimal ANC in the rural (28.0%) continue to SBA (Fig. 3). The proportion of continuation to PNC reduces by about 6 folds from 23.4 to 4.3% in urban and from 13.6% to 2.2% in rural. CoC completion rate in urban (4.3%) is almost twice the rural (2.2%) (Fig. 3).

### Stepwise forward regression analysis of CoC completion predictors

The stepwise logit model predictors of CoC completion are shown in Table 2. Woman’s age (*p*-value = 0.434), ethnicity (*p*-value = 0.617), pregnancy desire (*p*-value = 0.312), birth order (*p*-value = 0.437), health insurance (*p*-value = 0.265), medical permission (*p*-value = 0.512), child sex (p-value = 0.846) and child size(p-value = 0.860) and community illiteracy (p-value = 0.955) were not associated with maternity CoC completion at *p* < 0.10 and were therefore removed in the model (Table 2). Overall, 18 identified women factors that significantly increase in the log likelihood (Wald statistics ≥ 3) when added in turn at *p* < 0.10 were retained in the model (Table 2).

**Table 2:** Stepwise binary logistic regression of the predictors of maternity continuum of care completion.

Women characteristics	B	exp (B)	SE	Wald	*p*-value
Woman’s age	0.044	1.045	0.056	0.613	0.434
Education	0.201^a^	1.223	0.047	18.607	< 0.001
Marital status	0.316^c^	1.372	0.145	4.736	0.030
Partner education	0.181^a^	1.198	0.043	17.682	< 0.001
Religion	− 0.213^b^	0.808	0.072	8.712	0.003
Ethnicity	− 0.015	0.985	0.030	0.251	0.617
Wealth	0.207^a^	1.230	0.054	14.433	< 0.001
Media exposure	0.150^c^	1.162	0.069	4.701	0.030
Wanted pregnancy	0.063	1.065	0.063	1.022	0.312
Birth order	− 0.022	0.978	0.028	0.605	0.437
Health insurance	0.161	1.175	0.144	1.242	0.265
Medical permission	0.084	1.088	0.128	0.431	0.512
Medical help – money	− 0.130^d^	0.878	0.074	3.043	0.081
ANC provider	− 0.564^a^	0.569	0.049	132.475	< 0.001
Healthcare decider	0.083^c^	1.086	0.036	5.294	0.021
Iron drug taken	0.908^a^	2.479	0.123	54.139	< 0.001
TT taken	0.264^a^	1.302	0.057	21.577	< 0.001
Place of delivery	− 0.545^a^	0.580	0.075	52.535	< 0.001
Mode of delivery	0.521^a^	1.684	0.115	20.707	< 0.001
Child sex	0.012	1.021	0.061	0.038	0.846
Child size	− 0.006	0.994	0.036	0.031	0.860
Place of residence	− 0.233^b^	0.792	0.075	9.600	0.002
Geopolitical zone	0.147^a^	1.158	0.022	46.664	< 0.001
Distance to medical facility	0.121^d^	1.129	0.089	3.371	0.073
Rural proportion	− 0.242^b^	0.785	0.080	9.122	0.003
Community illiteracy	0.003	1.003	0.061	0.003	0.955
Community poverty	− 0.091^d^	0.913	0.047	3.733	0.053

*ANC* Antenatal Care, *CHEW* Community Health Extension Worker, *CHW* Community Health Worker, *TBA* Traditional Birth Attendants, *TT* Tetanus Toxoid, *CS* Caesarian Section. ^a^*p* < 0.001; ^b^*p* < 0.01; ^c^*p* < 0.05 ^d^*p* < 0.10.

### Multilevel predictors of maternity CoC completion among women (15–49 years)

The predictors of maternity CoC completion are shown in Table 3 (model I–IV). Model I is the null model with the constant term only. All the predictors group in the individual level model II are significant except for subgroup that receive 1 TT in pregnancy (*p* > 0.05), traditional religion (*p* > 0.05) and auxiliary ANC provider (*p* > 0.05) (Table 3). The community predictors in model III are only insignificant for the predictor group of the community poverty (*p* > 0.05). The full model IV that combined model I-III shows that odds of CoC completion increase with education and wealth (Table 3). Odds of CoC completion is 35% higher among married than unmarried and among women that received 2 doses of TT than those that receive 0 (AOR = 1.35; 95% CI 1.02–1.80). media exposure increases the likelihood by 22% (AOR = 1.22; 95 % CI 1.06–1.40). Women who received ANC from CHEW/CHW (AOR = 0.15; 95% CI 0.09–0.25) and TBA (AOR = 0.05; 95% CI 0.02–0.10) are less likely than those that received from doctors to complete the CoC respectively (Table 3). Women who took Iron drug (AOR = 1.84; 95% CI 1.43–2.35) and have CS delivery(AOR = 1.70; 95% CI 1.37–2.11) are almost twice as likely to complete the CoC. Place of residence, geopolitical zone and community rural proportion are significant (*p* < 0.05) community predictors (Table 3). The community level cluster variances and the ICC is large for the null model I and decreases as the factors are added in model II-IV. Thus, 28.5% of the CoC completion effect is attributable to the community predictors with 63.2% of the variation explained in the full CoC model compared to the null model. The median odds ratio shows that CoC completion is twice as likely in high-risk community compared to the low-risk community (Table 3).

**Table 3:** Multilevel Predictors of Maternity Continuum of Care Completion.

Characteristics	Model I(Null)		Model II(Individual)		Model III(Community)		Model IV(Full)	
	AOR(95% CI)	*p*-value	AOR(95% CI)	*p*-value	AOR(95% CI)	*p*-value	AOR(95% CI)	*p*-value
Individual-level								
Education								
No formal education			ref				Ref	
Primary			1.36 (1.06–1.74)	0.013			1.23 (0.96–1.59)	0.091
Secondary			1.52 (1.19–1.92)	0.001			1.34 (1.05–1.71)	0.017
Tertiary			1.84 (1.38–2.45)	< 0.001			1.61 (1.20–2.16)	0.001
Marital status								
Unmarried			ref				Ref	
Married			1.43 (1.08–1.90)	0.011			1.35 (1.02–1.80)	0.034
Partner education								
No formal education			ref				Ref	
Primary			1.93 (1.44–2.58)	< 0.001			1.81 (1.35–2.43)	< 0.001
Secondary			2.16 (1.64–2.83)	< 0.001			2.12 (1.61–2.78)	< 0.001
Tertiary			1.91 (1.42–2.57)	< 0.001			1.96 (1.46–2.64)	< 0.001
Religion								
Christianity/catholic			ref				Ref	
Islam			0.82 (0.70–0.97)	0.019			0.94 (0.78–1.12)	0.469
Traditional/other			0.78 (0.25–2.39)	0.675			0.81 (0.26–2.46)	0.706
Wealth								
Poor			ref				Ref	
Average			1.89 (1.51–2.36)	< 0.001			1.72 (1.37–2.15)	< 0.001
Rich			2.11 (1.67–2.66)	< 0.001			1.73 (1.35–2.21)	< 0.001
Media exposure								
No			ref				Ref	
Yes			1.26 (1.09–1.44)	0.001			1.22 (1.06–1.40)	0.004
Medical help-money								
Not big problem			ref				Ref	
Big problem			0.86 (0.76–0.98)	0.031			0.84 (0.73–0.97)	0.024
ANC provider								
Doctor			ref				Ref	
Nurse/midwifery			0.58 (0.49–0.69)	< 0.001			0.59 (0.50–0.71)	< 0.001
Auxiliary nurse/midwifery			1.08 (0.77–1.51)	0.647			1.02 (0.72–1.43)	0.901
CHEW/CHW			0.15 (0.09–0.24)	< 0.001			0.15 (0.09–0.25)	< 0.001
TBA/no one			0.05 (0.02–0.10)	< 0.001			0.05 (0.02–0.10)	< 0.001
Healthcare decider								
Partner			ref				Ref	
Respondent			1.50 (1.24–1.81)	< 0.001			1.37 (1.13–1.66)	0.001
Joint decision			1.21 (1.04–1.39)	0.011			1.12 (0.96–1.29)	0.143
Iron drug taken								
No			ref				Ref	
Yes			1.78 (1.39–2.28)	< 0.001			1.84 (1.43–2.35)	< 0.001
TT taken								
None			ref				Ref	
1			0.96 (0.71–1.30)	0.805			0.92 (0.68–1.26)	0.628
2 +			1.40 (1.06–1.84)	0.016			1.35 (1.02–1.78)	0.030
Place of delivery								
Home			ref				Ref	
Health facility			0.60 (0.52–0.70)	< 0.001			0.54 (0.47–0.63)	< 0.001
Mode of delivery								
Vaginal delivery			ref				Ref	
Delivery by CS			1.72 (1.38–2.12)	< 0.001			1.70 (1.37–2.11)	< 0.001
Community-level								
Place of residence								
Urban					ref		Ref	
Rural					0.52 (0.42–0.63)	< 0.001	0.81 (0.67–0.98)	0.036
Geopolitical zone								
North central					ref		Ref	
Northeast					0.65 (0.46–0.93)	0.017	1.05 (0.75–1.48)	0.746
Northwest					0.47 (0.34–0.66)	< 0.001	0.80 (0.57–1.11)	0.185
Southeast					2.10 (1.52–2.90)	< 0.001	1.59 (1.15–2.18)	0.004
South-south					1.79 (1.27–2.51)	0.001	1.30 (0.92–1.81)	0.127
Southwest					2.48 (1.81–3.38)	< 0.001	1.91 (1.42–2.56)	< 0.001
Distance to medical facility								
Not a big problem					ref		Ref	
Big problem					0.68 (0.58–0.81)	< 0.001	1.03 (0.86–1.24)	0.702
Rural proportion								
Low					ref		Ref	
High					0.72 (0.57–0.92)	0.007	0.78 (0.63–0.98)	0.032
Community Poverty								
Low					ref		Ref	
Average					1.00 (0.80–1.26)	0.958	0.94 (0.75–1.17)	0.589
High					0.89 (0.69–1.15)	0.381	0.91 (0.72–1.15)	0.439
Constant	0.04 (0.03–0.04)	< 0.001	0.01 (0.00–0.02)	< 0.001	0.07 (0.05–0.10)	< 0.001	0.02 (0.01–0.03)	< 0.001
Random effects parameters								
Variance (95% CI)	1.77 (1.48–2.12)		0.70 (0.55–0.87)		0.96 (0.78–1.17)		0.65 (0.52–0.82)	
ICC (95% CI)	51.9 (47.5–56.3)		29.8 (25.4–34.6)		36.8 (32.3–41.6)		28.5 (24.1–33.4)	
MOR (95% CI)	3.54 (3.17–3.99)		2.21 (2.02–2.43)		2.54 (2.31–2.79)		2.15 (1.98–2.36)	
PCV (%)	ref		60.5 (0.58–0.62)		45.7 (44.8–47.3)		63.2 (61.3–64.9)	
Model fit statistics								
− 2LogL	8480.38		7478.96		8128.04		7416.90	
AIC	8484.38		7530.96		8152.03		7488.89	
BIC	8500.12		7735.53		8246.45		7772.14	
Sample size								
Individual	19,474		19,474		19,474		19,474	
Community	1400		1400		1400		1400	

*ANC* Antenatal Care, *CHEW* Community Health Extension Worker, *CHW* Community Health Worker, *TBA* Traditional Birth Attendants, *TT* Tetanus Toxoid, *CS* Caesarian Section, #reference group.

### Assessment of the maternity CoC model adequacy

The fit statistics of the maternity CoC model is presented in Table 3. The deviance statistics (− 2LL) computed for each of the specified model I–IV are 8480.38, 7478.96, 8128.04 and 7416.90 respectively. The full model-IV (mixed-effect) has the least deviance (7416.90) (Table 3). Similarly, the values of the Akaike and Bayes information criteria (AIC = 7488.89; BIC = 7772.14) for the full model-IV is the least of all the models and therefore perform best (Table 3).

### Subnational distribution of maternity continuum of care completion

**Figure 4:**
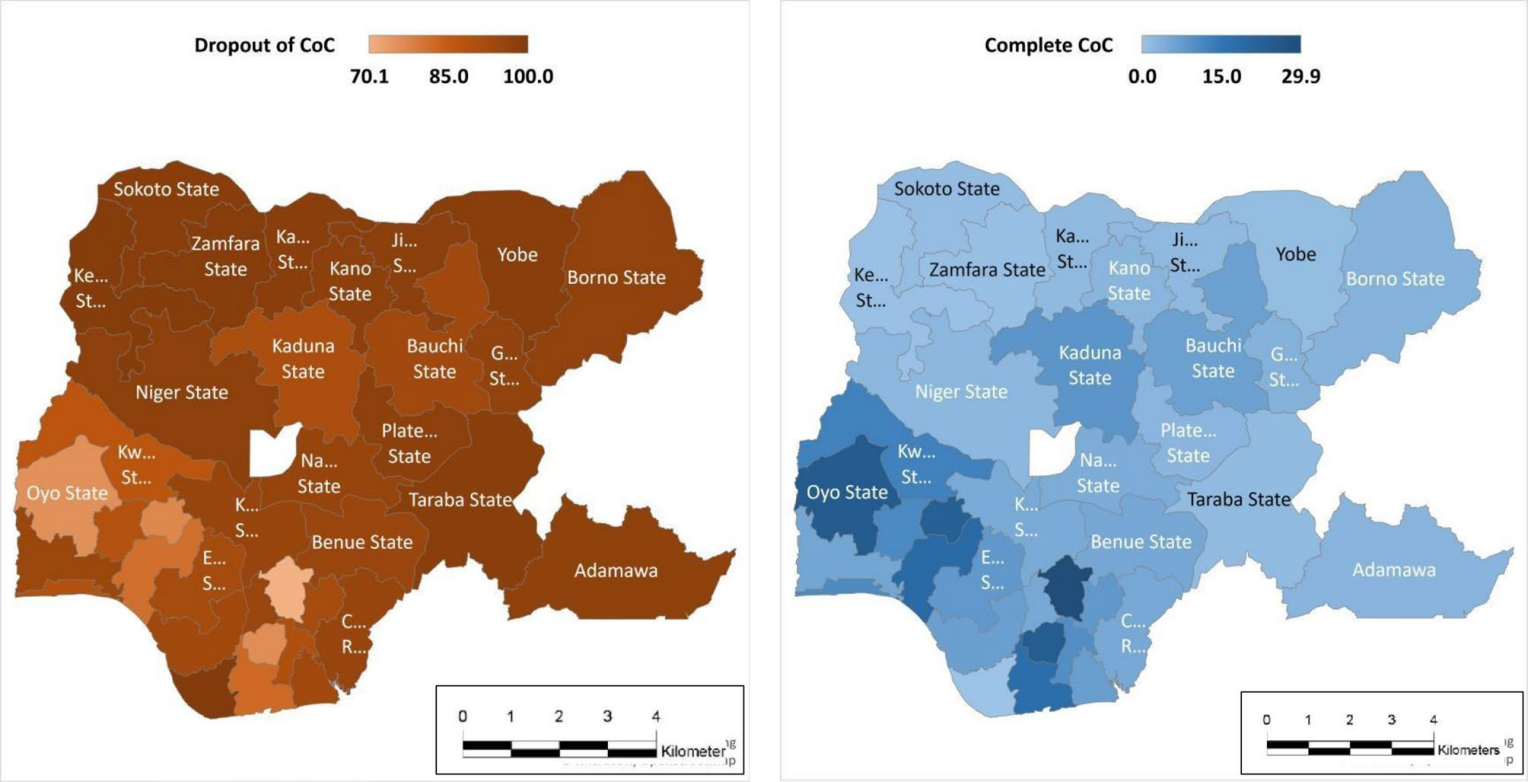
Subnational Distribution of Dropout and Completion of Maternity Continuum of Care in Nigeria (generated from Microsoft OpenStreetMap).

Figure 4 shows the distribution of maternity continuum of care completion by states. Completion rate is higher in Southern region “Enugu (29.9%), Oyo (24.5%), Imo (24.0%), Ekiti (22.8%)” than in Northern region “Kebbi (0.4%), Zamfara (0.7%)” among others states where there are more dropouts (Fig. 4). Also, very high proportion of Women dropout from the CoC completion in Sokoto (98.9%), Yobe (98.8%), Taraba (98.4%), Katsina (98.3%), Jigawa (98.1%) (Fig. 4).

### Subnational analysis of maternity continuum of care completion

Table 4 present the state proportion and effect on maternity CoC completion relative to the largest (1620, 8.32%) women population in Kano state. Of the 6.52% (1269/19,474) maternity CoC completion prevalence in Nigeria, CoC completion was highest in Oyo (0.80%) followed by Kaduna (0.54%), Rivers (0.45%), Imo (0.43%), Lagos (0.42%) and Enugu (0.42%), while 0% maternity CoC completion was seen in Bayelsa (Table 4). CoC completion was near 0% in Kebbi (0.02%), Sokoto (0.03%) and Zamfara (0.03%) (Table 4). Compared to Women in Kano, Enugu women are about 13.5 times more likely to complete the maternity CoC (OR = 13.48, 9.32–19.49) (Table 4). Women in Ekiti (OR = 10.84, 7.21–16.28), Imo (OR = 10.64, 7.37–15.37) and Oyo ((OR = 10.47, 7.46–14.67) are more than 10 times more likely to Complete the maternity CoC than those in Kano (Table 4). Whereas the odds of completing maternity CoC reduces by 80%, 69% and 67% in Kebbi (OR = 0.20, 0.06–0.57), Zamfara (OR = 0.31, 0.13–0.83) and Sokoto (OR = 0.33, 0.13–0.70) respectively (Table 4). Odds of maternity CoC completion was also high by more than 5 folds in Ondo, Rivers and Kwara states when compared to Kano (Table 4).

**Table 4:** Subnational impact on maternity continuum of care completion in Nigeria.

States	Maternity CoC completion (ANC4 + SBA + PNC)					
	Incomplete CoC n (%)	Complete CoC n (%)	All n (%)	OR exp (β)	95% CI	*p*-value
#Kano	1577 (8.10)	43 (0.22)	1620 (8.32)	Ref		
Sokoto	557 (2.86)	5 (0.03)	562 (2.89)	0.33*	0.13–0.83	0.019
Zamfara	765 (3.93)	6 (0.03)	771 (3.96)	0.31**	0.13–0.70	0.005
Katsina	1286 (6.60)	20 (0.10)	1306 (6.71)	0.57*	0.33–0.97	0.038
Jigawa	858 (4.41)	19 (0.10)	877 (4.50)	0.80	0.46–1.37	0.411
Yobe	692 (3.55)	8 (0.04)	700 (3.60)	0.44*	0.21–0.93	0.031
Borno	667 (3.43)	25 (0.13)	693 (3.56)	1.38	0.84–2.26	0.193
Adamawa	452 (2.32)	12 (0.06)	464 (2.38)	0.97	0.51–1.84	0.935
Gombe	391 (2.01)	12 (0.06)	403 (2.07)	1.07	0.56–2.05	0.828
Bauchi	804 (4.13)	57 (0.29)	860 (4.42)	2.52***	1.69–3.74	< 0.001
Kaduna	1262 (6.48)	105 (0.54)	1367 (7.02)	2.96***	2.07–4.22	< 0.001
Kebbi	695 (3.57)	4 (0.02)	699 (3.59)	0.20**	0.06–0.57	0.003
Niger	779 (4.0)	16 (0.1)	795 (4.08)	0.76	0.43–1.35	0.350
FCT Abuja	109 (0.56)	15 (0.08)	124 (0.64)	4.73***	2.62–8.53	< 0.001
Nasarawa	283 (1.45)	14 (0.07)	297 (1.53)	1.82	0.99–3.30	0.051
Plateau	323 (1.66)	10 (0.05)	333 (1.71)	1.13	0.56–2.25	0.726
Taraba	426 (2.19)	6 (0.03)	432 (2.22)	0.53	0.22–1.23	0.141
Benue	535 (2.75)	32 (0.16)	567 (2.91)	2.16**	1.36–3.41	0.001
Kogi	251 (1.29)	13 (0.07)	264 (1.36)	1.85	0.99–3.45	0.052
Kwara	291 (1.50)	45 (0.23)	336 (1.73)	5.35***	3.52–8.12	< 0.001
Oyo	470 (2.41)	154 (0.80)	624 (3.2)	10.47***	7.46–14.67	< 0.001
Osun	347 (1.78)	41 (0.21)	388 (1.99)	4.16***	2.71–6.38	< 0.001
Ekiti	148 (0.76)	50 (0.26)	198 (1.02)	10.84***	7.21–16.28	< 0.001
Ondo	204 (1.05)	46 (0.23)	250 (1.28)	7.48***	4.93–11.34	< 0.001
Edo	192 (0.99)	21 (0.11)	213 (1.09)	3.74***	2.20–6.32	< 0.001
Anambra	514 (2.64)	30 (0.15)	544 (2.79)	2.09**	1.31–3.33	0.002
Enugu	186 (0.95)	82 (0.42)	268 (1.37)	13.48***	9.32–19.49	< 0.001
Ebonyi	380 (1.95)	34 (0.17)	414 (2.12)	3.14***	2.00–4.93	< 0.001
Cross river	157 (0.81)	9 (0.05)	166 (0.85)	2.05	0.99–4.21	0.051
Akwa Ibom	266 (1.36)	22 (0.11)	288 (1.48)	2.99***	1.79–4.98	< 0.001
Abia	180 (0.92)	20 (0.10)	200 (1.48)	3.98***	2.34–6.73	< 0.001
Imo	252 (1.29)	84 (0.43)	336 (1.72)	10.64***	7.37–15.37	< 0.001
Rivers	432 (2.22)	88 (0.45)	520 (2.67)	6.82***	4.73–9.82	< 0.001
Bayelsa	102 (0.52)	0 (0.00)	102 (0.52)	1.00	–	–
Delta	297 (1.53)	19 (0.10)	316 (1.62)	2.28**	1.32–3.91	0.003
Lagos	738 (3.79)	81 (0.42)	819 (4.21)	3.85***	2.66–5.57	< 0.001
Ogun	336 (1.73)	21 (0.11)	357 (1.83)	2.18**	1.28–3.69	0.004
Total	18,205 (93.48)	1269 (6.52)	19,474 (100.00)			

^#^Reference category; ***significant at *p* < 0.001; ** significant at *p* < 0.01; *significant at *p* < 0.05.

## Discussion

This study applied the multilevel analysis techniques to investigates the individual and community prevalence and predictors as well as the subnational distribution of the maternity CoC completion ‘from pregnancy to childbirth and post-delivery i.e., ANC, SBA and PNC’ based on the WHO recommendations for optimal health and survival of mother and newborn. This is to achieve an evidence-based policy strategy specific to supporting and improving MNCH programming at the community and subnational level and, towards achieving the 2030 SDG-3 goal.

It was observed that more than two-third of the pregnant women in Nigeria attended ANC, but less than three-fifths (57.4%) completed the minimum recommended four visits. This is similar to national survey report and studies on ANC compliance with WHO recommendation in Nigeria^12,16^. Prevalence of SBA continuation after optimal ANC uptake is 37% and completion rate of the three essential maternity CoC (i.e., ANC SBA and PNC) is only 6.5%. However, nearly two-third of the prevalence of maternity CoC completion in Nigeria was observed in the urban compared to the one-third in the rural. Which can be ascribed to the easier access to maternal healthcare in urban compared to rural and hence the higher likelihood of maternity CoC completion in urban than the rural as reported in the recent multi-country study in SSA^52^.

Overall, prevalence of continuation of care diminishes as women progress from pregnancy to delivery and post-delivery with significant dropout observed at the PNC stage. This is because those that utilized SBA tends to be absent from PNC as they are less likely to have post-delivery complications compared to those that do not utilize SBA at delivery and thus, high dropout rate was observed at PNC as found in study investigating dropout pattern in Nigeria and other SSA countries^20,22,53^. Also, completion rate is cumulatively higher in southern states than northern states owing to contribution of Oyo with highest prevalence of maternity CoC completion.

Education, residence, wealth, decider of healthcare, tetanus toxoid taken in pregnancy, place of residence, distance to medical facility, geopolitical zone, among other individual and community-level factors were significantly related to maternity continuum of care. Child sex and community illiteracy level were however not associated with women completion of CoC. Comparable factors were reported in studies in SSA to be associated with maternity CoC completion^2,4,22,37,52,54^.

It was discovered that odds of gamut of maternity care uptake, continuation and completion increase with progression in education and wealth. Maternal education effect is understandably due to exposure and ability to listen, read and write as women with higher education are more likely to be able to comprehend and follow medical instructions and prescriptions than those with secondary education^39^, and those with secondary education perform better in maternal service utilization than those with primary education only and so on. Agreeing with recent findings from studies on the effect of female education and moderating role of partner education on maternal healthcare in SSA^55,56^. The effect of wealth is attributed to being able to bear expenses of maternity range of cares as commitment can be motivated by financial strength and it is not surprising that women who had the big problem of getting money for medical help are less likely to complete the maternity gamut of care. This is in congruent with study outcome on positive effect of wealth and health insurance coverage on maternal healthcare in SSA^57,58^.

Women exposure to media and healthcare decision making positively influence maternity CoC completion by 22% and 37% respectively. This explains the role of media (listening to radio, watching television, and reading newspapers) as well as the women autonomous health decision making on their own health compared to partner decision as only pregnant women knows her health better. Similar findings were reported from studies on factors associated with maternity healthcare service utilization and CoC completion in African settings^18,34,36,37,39,52,54^.

Furthermore, the chance of completing maternity CoC is higher in women who had a minimum of two required number of tetanus injection than those that had none. Since the WHO recommended 2 or more TT immunization required for women to develop antibodies to protect their newborn against tetanus and will encourage more ANC visits to facilitate optimal reception of ANC and subsequently drives SBA and PNC as previously enunciated to have a big impact on infant^59^. Also, Iron drug taken in pregnancy almost twice increase the likelihood of a woman completing the CoC compared to those that do not take iron supplement in pregnancy. This was corroborated by the findings from previous study on maternity predictors of CoC coverage^22^.

However, having nurses, TBA and CHEW/CHW as ANC provider, big problem of getting money for medical help and health facility delivery are the individual-level predictors with negative influence on the maternity CoC completion. Hence women whose ANC was provided by CHEW and TBA are about 7 and 20 times less likely to complete the maternity CoC respectively. Having hospital birth almost twice reduces the likelihood of CoC completion as those who had hospital births after ANC are more likely to have vaginal birth and dropout of PNC. This is in consonance with findings on dropouts of CoC, predictors of maternity CoC in Nigeria and the determinants of maternity CoC in Cambodia^20,22,46,60^. Also, Caesarian section delivery positively drive CoC completion after ANC utilization as a result of the necessity to seek for medical help after delivery as women who had CS birth are more likely to attend PNC for follow-up care on surgical site due to poor postnatal quality of life which adversely impacted the optimality of newborn breastfeeding compared to women who had vaginal birth^61,62^.

It was observed that place of residence, geopolitical zone and community rural percentage are the Community-level factors associated with maternity CoC completion in Nigeria. Thus, women who resides in the rural and within community of high rural population proportion have 23% and 28% excess risk of not completing the maternity CoC. Which can be ascribed to the prevailing problem of limited access to health facility majorly worsened by distance, health finances and socio-cultural factors in the rural communities as substantiated by recent studies in SSA^22,46,52^. Nevertheless, women in the southern (south–south, southeast, and southwest) region are (30%, 59% and 91%) more likely to complete the maternity CoC than their counterpart in the north.

The significance of Community-level variance indicated the suitability of the multilevel analysis to evaluate dependency in maternity CoC completion. The ICC implies that 28.5% of the variation in maternity CoC completion are attributable to the community heterogeneity. Thus, 63.2% of the variation in maternity CoC completion were explained by the community-level effect relative to the null model. Corresponding findings were observed in studies applying multilevel method to evaluate predictors of maternity CoC dropout and completion^20,48,63^. The model assessment based on deviance statistics further stressed the robustness of the multilevel method and identified the full model with random and fixed effect (mixed effect) component as the optimal model across the four specified model “Model I–IV”. Studies on multilevel analysis also reported findings analogous to comparison among null, random, fixed and the mixed effect models^50,51^.

When the subnational impact on maternity CoC completion was compared with the state with largest women population (Kano), Women in Enugu state reported highest effect and are about 13 times more likely to complete the maternity CoC. Also, Ekiti, Imo and Oyo women are more than 10 times as likely to achieve maternity CoC completion. The chance of maternity CoC completion is about 7 times more in Ondo and Rivers than Kano. Also, the subnational effect was positive in Kwara, FCT, Osun, Abia, Lagos, Edo, Akwa-Ibom, Kaduna, Bauchi, Delta, Ogun, Benue, and Anambra. However, the effect was negative in Kebbi, Zamfara, Sokoto, Yobe and Katsina as pregnant women in these states are 2–5 times less likely to complete the maternity range of cares.

### Strengths and limitations

The study was not free from recall bias majorly associated with cross-sectional studies as it relies on respondent’s events in the last 5 years preceding the survey. Inference from the study should be interpreted based on association not causal factor as it does not control for temporality and other condition to ascertain causality. Analysis was also subjected to the operationalized variables measured in the DHS based on the survey questionnaire as information such as type of healthcare facility (primary, secondary, and tertiary) were not collected. However, the use of nationally representative large dataset improves the target population generalizability. Also, the application of sampling weight further enhanced the accuracy and reliability of the study findings therein. The multilevel analysis technique that accounted for clustering effect by assessing variations between clusters was suitable for the cluster survey design and can also be regarded as strength.

## Conclusions

In conclusion, only one in fifteen pregnant women completed the maternity continuum of care. Completion rate is however significantly different between communities, with the two-thirds of the prevalence in urban and only one-third in the rural. Women CoC completion is twice more likely in the urban clusters than the rural and thus highlight place of residence, community rural proportion and geopolitical zone as the community drivers of the maternity gamut of care completion. Chance of CoC completion increase by education and wealth but decrease by cadre of ANC provider. Women autonomy to her own healthcare decision, media exposure, tetanus toxoid and iron drug taken in pregnancy positively influence CoC completion while health facility delivery and medical finances negatively influence CoC completion. About One-third of the variation in maternity CoC completion is attributable to the significant community-level factors. Hence the mixed-effect model was optimal. Women CoC Completion rate is higher in southern than northern sub-nationals, with Enugu, Ekiti, Imo and Oyo the four topmost states with highest likelihood of maternity CoC completion.

### Recommendations

The generally low coverage of maternity CoC completion is an indication for the need to strengthen maternity healthcare practice through sensitization programs, capacity buildings and increase access to quality maternal healthcare service particularly in the rural. ANC packages should be reviewed to encourage optimal visits and discourage dropouts. Northern sub-nationals should emulate what works and learn from what doesn’t work in the Southern region. Contextual research on dropout of maternity CoC completion is required particularly in the rural to understand the militating socio-cultural factors towards devising interventional programming strategy.

## Data Availability

The de-identified data is available in the public domain . Analyzed dataset used in this current study are available on reasonable request from the corresponding author and can be access at the open repository of the DHS program www.dhsprogram.com.

## Abbreviations

ANC: Antenatal care
CI: Confidence interval
CoC: Continuum of care
ICC: Intra-cluster correlation
IMR: Infant mortality rate
LMIC: Lower–middle-income country
MMR: Maternal mortality ratio
MNCH: Maternal newborn and child health
NDHS: Nigerian Demographic and Health Survey
NMR: Neonatal mortality rate
PNC: Postnatal care
PRMR: Pregnancy related mortality ratio
SBA: Skilled birth attendants
SDG: Sustainable Development Goal
SSA: Sub Saharan Africa
U5MR: Under 5 mortality rate
WHO: World Health Organization
MOR: Median odds ratio
PCV: Proportional change in variance
CS: Caesarian section
CHEW: Community health extension worker
CHW: Community health worker
TBA: Traditional birth attendants
AOR: Adjusted odds ratio
